# Acetylated Histones in Apoptotic Microparticles Drive the Formation of Neutrophil Extracellular Traps in Active Lupus Nephritis

**DOI:** 10.3389/fimmu.2017.01136

**Published:** 2017-09-14

**Authors:** Nils Rother, Elmar Pieterse, Jelle Lubbers, Luuk Hilbrands, Johan van der Vlag

**Affiliations:** ^1^Department of Nephrology, Radboud Institute for Molecular Life Sciences, Radboud University Medical Center, Nijmegen, Netherlands

**Keywords:** neutrophil extracellular traps, microparticles, lupus nephritis, acetylated histones, systemic lupus erythematosus, NETosis

## Abstract

**Objective:**

Systemic lupus erythematosus (SLE) is an autoimmune disease characterized by the presence of autoantibodies against nuclear components. Lupus nephritis (LN) is the major cause of morbidity and mortality in patients with SLE. Central to the pathogenesis of SLE is the accumulation of cellular waste, especially apoptotic microparticles (MPs), which stimulates diverse immune reactions including the formation of neutrophil extracellular traps (NETs). In this study, we investigated the content of MPs from SLE patients with and without (active) LN, their capacity to stimulate NET release, and assessed the molecular mechanisms underlying MP-induced NETosis.

**Methods:**

MPs from SLE patients with biopsy-proven active LN, remissive LN, without LN, and healthy controls were characterized by flow cytometry. Isolated neutrophils were exposed to MPs derived from either patient plasma or apoptotic human umbilical vein endothelial cells, and NET release was quantified by immunofluorescence imaging, spectrofluorometry or an in-house developed NET ELISA.

**Results:**

MPs from SLE patients with active LN contain higher levels of acetylated chromatin compared to MPs from those with remissive LN, without LN, or healthy controls. MPs enriched in hyperacetylated chromatin are more potent in inducing NETosis when compared to MPs containing moderate acetylated chromatin. The release of NETs in response to MPs occurs rapidly in a concentration-dependent manner and proceeds independent from the formation of reactive oxygen species (ROS).

**Conclusion:**

Our data suggest that MPs containing acetylated chromatin drive ROS-independent NET release in SLE patients with active LN, which may lead to the glomerular deposition of NETs and subsequent NET-driven LN.

## Introduction

Systemic lupus erythematosus (SLE) is an autoimmune disease characterized by the presence of autoantibodies against nuclear antigens, such as nucleosomes, DNA, and histones (1). Immune complexes of autoantibodies and chromatin form depositions and trigger serious inflammatory conditions in multiple organs, including lupus nephritis (LN) in the kidneys. LN is a major cause of morbidity and mortality in patients with SLE and causes proteinuria and a gradual decline of kidney function (2–4). During the disease course, states of quiescent disease can alternate with periods of active disease, the so-called flares. Although the triggers of these flares are largely unknown, they lead to increased production of autoantibodies and enhancement of inflammatory processes (5–7).

The presence of nuclear antigens in the circulation of patients with SLE can be explained by increased apoptosis and/or an impaired clearance of apoptotic cells. Apoptotic cell-derived constituents within the extracellular space trigger immune reactions by innate and adaptive immune cells (8–11). Apoptotic cells can give rise to the formation of microparticles (MPs), which are small vesicles generally defined as <1 μm in size, that carry a range of immunostimulatory proteins, including apoptosis-modified chromatin enriched in methylated and particularly acetylated histone residues (12–14). MPs have been identified and characterized in plasma of SLE patients and were previously shown to be highly pro-inflammatory (15–18). Autoantibodies against these apoptosis-associated histone modifications can be found in patients with SLE, suggesting that these modifications play a role in breaking the immune tolerance (5, 19–21).

In addition to apoptosis, abnormalities in the formation and degradation of neutrophil extracellular traps (NETs) have been linked to the autoimmune response in SLE and LN (22). NETs are formed during a specialized cell death program of neutrophils, termed NETosis and are generally described as web-like structures composed of DNA and antimicrobial proteins, e.g., myeloperoxidase (MPO) and neutrophil elastase (NE) (23). Although being essential for antimicrobial defense, NETs can also cause tissue damage and evoke autoimmune responses, when persistently present in the extracellular space (24, 25).

Recently, we showed that MPs from patients with SLE but not MPs from patients with other autoimmune diseases can activate blood-derived dendritic cells and induce the formation of NETs (15). However, the reason for the increased immunogenicity of MPs from patients with SLE remained unanswered. Moreover, the mechanisms by which MPs induce NET formation remained to be established. In this study, we investigated the mechanisms and clinical relevance of MP-induced NETosis in SLE.

## Materials and Methods

### Human Material

Blood samples were collected from SLE patients and healthy controls that either participated in the Dutch SLE Nephritis Study (26) or in subsequent studies at the Radboud university medical center, The Netherlands. All patients fulfilled at least four American College of Rheumatology criteria for SLE. Patients were either defined as patients with (i) active LN, (ii) remissive LN, or (iii) without LN. Patients with active LN were defined as having glomerular hematuria or proteinuria of ≥0.5 g/24 h and LN was confirmed by renal biopsy. Patients with active LN were longitudinally followed and new blood was retrieved at the time proteinuria was ≤0.5 g/24 h and/or hematuria had disappeared (remissive LN). Patients with LN were treated with cyclophosphamide or a combination of azathioprine and methylprednisolone pulses, both combined with oral prednisone until reaching criteria for remission. Blood samples of patients categorized as active LN were collected before the start of treatment. Clinical parameters, such as C3 levels, anti-dsDNA titers, creatinine, and proteinuria, and SLE disease activity index [SLEDAI (27)] were determined in our diagnostic facility by routine measurements (Table S1 in Supplementary Material). The collection of blood samples was approved by the ethics committees of all participating hospitals and the study has been carried out in compliance with the Helsinki declaration. Written informed consent was obtained from all subjects.

### Isolation of MPs

Blood was centrifuged for 10 min at 3,000 × *g* and platelet-poor plasma was stored at −80°C until further use. For the isolation of plasma-derived MPs, plasma was diluted in PBS and centrifuged for 5 min at 500 × *g* at 4°C. Subsequently, the supernatant was centrifuged 10 min at 20,800 × *g* at 4°C.

### *In Vitro* Generation of Endothelial Cell-Derived Apoptotic MPs

Human umbilical vein endothelial cells (HUVECs) were maintained in fibronectin-coated Corning cell culture flasks (Sigma-Aldrich) in EGM-2-medium supplemented with 2% FCS and EGM-2 SingleQuots (Lonza) and cultured with 5% CO_2_ at 37°C. Apoptosis in HUVECs was induced by 10 µM 4 Nitroquinoline 1 oxide (4-NQO; Sigma-Aldrich) for 24 h. Culture supernatants were then centrifuged (5 min, 500 × *g*, 4°C) followed by an additional centrifugation step (10 min, 20,800 × *g*, 4°C) to pellet endothelial cell-derived MPs. Where indicated, HUVECs were pretreated with the histone deacetylase inhibitor trichostatin A (TSA) (Sigma-Aldrich) for 24 h prior to the induction of apoptosis to generate MPs enriched in hyperacetylated chromatin.

### Flow Cytometry

Isolated MPs were washed with PBS containing 0.1% bovine serum albumin (PBA), and incubated for 30 min at 4°C with the monoclonal anti-histone antibodies KM-2 [anti-H4K8,12,16Ac (12)] and LG11-2 [anti-H2BK12Ac (13)] or their appropriate isotype controls (UPC-10; Sigma). MPs were subsequently incubated for 30 min at 4°C with Alexa Fluor 488-conjugated goat anti-mouse IgG (Thermo Fisher Scientific). Finally, MPs were extensively washed and resuspended in PBA and analyzed using a FC500 flow cytometer (Beckman Coulter). For determination of MP concentrations, AccuCheck calibrated beads were added according to the manufacturer’s protocol (Thermo Fisher Scientific).

### Isolation of Neutrophils and Induction of NETosis

Neutrophils were isolated by Ficoll density gradient centrifugation using Lymphoprep™ (Stemcell Technologies), as described previously (28). Isolated neutrophils (3 × 10^5^ cells per cm^2^) were seeded in serum-free DMEM/F12 (Sigma-Aldrich) and exposed to MPs at indicated concentrations. Addition of 100 nM PMA (Sigma-Aldrich) or activated platelets to neutrophil cultures served as positive controls for NETosis induction. Platelets were isolated and activated with lipopolysaccharide (LPS, O111:B4, Sigma-Aldrich), as described previously (29). Where indicated, neutrophils were pretreated for 1 h with the ROS-inhibitor diphenyleneiodonium (40 µM, DPI, Enzo Life Sciences) prior to the addition of MPs or PMA.

### Quantification of NETs

After stimulation of neutrophils, adherent NETs were isolated by partially digesting the NETs in DMEM/F12 medium (Life Technologies) supplemented with 5 U/ml micrococcal nuclease (Worthington Biochemical Corporation). NET-derived DNA was incubated with 200 nM Sytox Orange (Thermo Fisher Scientific) and detected using a Tecan Infinite 200 multimode reader (Tecan industries). For the in-house developed NET ELISA, soluble NET fragments were captured using an anti-dsDNA antibody [antibody #36, mouse IgG2a, 1:4,000 (30)], blocked with 2% fish gelatin (Sigma-Aldrich), and detected with an anti-MPO antibody (mouse IgG1, 1:6,000, Biolegend). A horseradish peroxidase conjugated antibody (goat anti-mouse IgG1, 1:5,000, Jackson Immunoresearch) was used to detect the anti-MPO antibody. TMB substrate (Biolegend) was added according to the manufacturer’s instructions for 12 min. The reaction was stopped by adding 2 M sulfuric acid and absorbance was measured at 450 nm using a Spectophotometer (Benchmark Plus, Biorad).

### Immunofluorescence Microscopy

Purified neutrophils (10 × 10^5^ cells per cm^2^) were seeded in slide-flask chambers (Thermo Scientific) and exposed to MPs. After stimulation, cells and NETs were fixed in 4% paraformaldehyde (15 min, 4°C), and slides were stained for DNA (Sytox Orange; 200 nM), NE (1:200, Abcam), and/or MPO (1:400, Biolegend). Goat anti-rabbit and goat anti-mouse antibodies labeled with Alexa Fluor 488 (1:200, Thermo Fisher Scientific) were used to detect anti-NE and anti-MPO, respectively. Slides were embedded in Vectashield Mounting Medium (Brunschwig Chemie), and pictures were obtained with a Zeiss fluorescence microscope with Axiovision soſtware.

### DNase Activity

DNA degradation was assessed according to the protocol of Hakkim et al. (22) with slight modifications. Briefly, NET-associated DNA or calf thymus-derived DNA was adjusted to a concentration of 500 ng/ml and incubated with 10% recalcified plasma. After 180 min of incubation at 37°C, the remaining DNA was quantified with 200 nM Sytox Orange using fluorometry.

### Statistical Analyses

Data are reported as the means ± SEM of at least three experiments. Significance was determined by the appropriate statistical tests as specified in the figure legends. Correlation was analyzed using Spearman’s correlation coefficient. GraphPad Prism software (Version 5.03) was used for statistical testing. *p* values less than 0.05 were considered as statistically significant.

## Results

### MPs from Patients with Active LN are Enriched in Acetylated Chromatin

**Figure 1:**
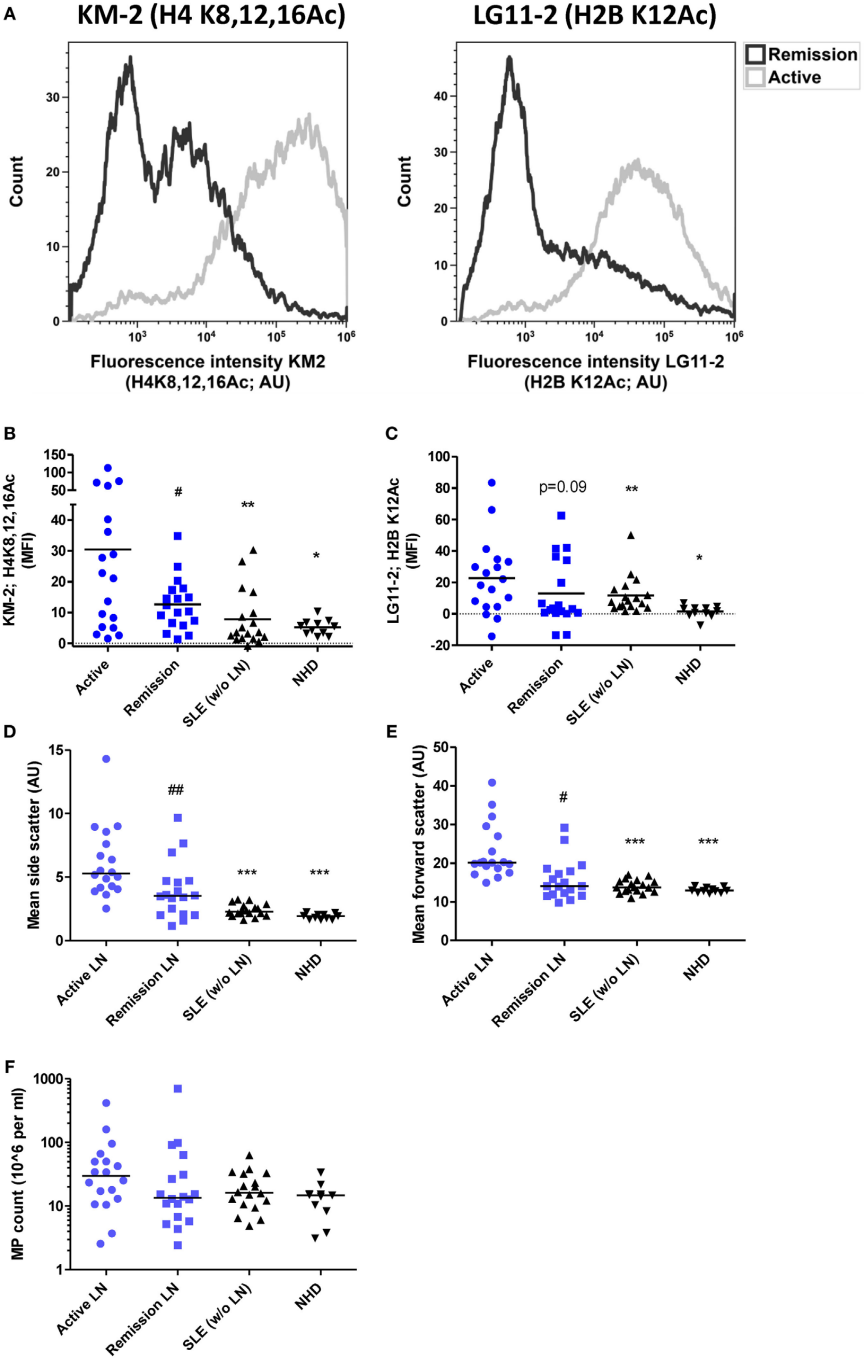
Degree of acetylation of histones in microparticles (MPs) isolated from patients with systemic lupus erythematosus (SLE) is increased during active lupus nephritis (LN). **(A)** Representative flow cytometer plots of one patient showing MPs stained for acetylated histone H4K8,12,16 (left panel; stained with monocolonal antibody KM-2) and acetylated histone H2BK12 (right panel; stained with monoclonal antibody LG11-2) during active and remissive period of disease. **(B,C)** Mean fluorescence intensity of MPs stained for H4K8,12,16Ac **(B)** and H2BK12Ac **(C)** corrected for isotype staining from patients with active LN (*n* = 18) compared to patients with LN at remission (*n* = 18) or patients without LN (*n* = 18) and normal healthy controls (NHD; *n* = 11). **(D,E)** Mean side and forward scatter of unstained MPs. **(F)** MP count in plasma as determined using counting beads. Data are presented for each patient separately with indication of the median of the groups. *p < 0.05, **p < 0.01, ***p < 0.001 compared to active disease patients, determined by Kruskal–Wallis test followed by Dunn’s *post hoc* test. #*p* < 0.05, ##*p* < 0.01 compared to active disease patients by paired Student’s *t* test **(B)** and Wilcoxon matched-pairs signed rank test in **(C–E)**.

We have previously described that SLE patients-derived MPs are capable of enhancing NETosis in neutrophils (15). Since aberrant NETosis has been particularly associated with the development of LN in SLE, we explored whether MPs from SLE patients with active LN differ and have a different NETosis-inducing capacity when compared to MPs from SLE patients with remissive LN or without LN. Therefore, we isolated MPs from the same patients at different time points, during active LN and after reaching remission. MPs isolated from patients during active LN showed an increased reactivity with the anti-histone antibodies KM-2 and LG11-2, targeting apoptosis-induced acetylated histone H4 (at K8,12,16) and H2B (at K12), respectively, when compared to MPs from the same patients during remissive LN or patients without LN (Figures 1A–C). Furthermore, MPs from patients with active LN showed a higher forward and side scatter compared to MPs from patients with remissive LN or without LN (Figures 1D,E), suggesting that these MPs originate from apoptotic cells, since they are generally larger in size and have more content compared to MPs derived from resting or activated cells (31). Importantly, the total number of MPs did not significantly differ between all groups (Figure 1F). In summary, circulating MPs in patients with active LN are relatively large and have more apoptosis-associated chromatin modifications, which decline during the remission of LN and are even lower in patients without LN.

### HUVEC-Derived MPs Induce NETosis in a Concentration-Dependent Manner

**Figure 2:**
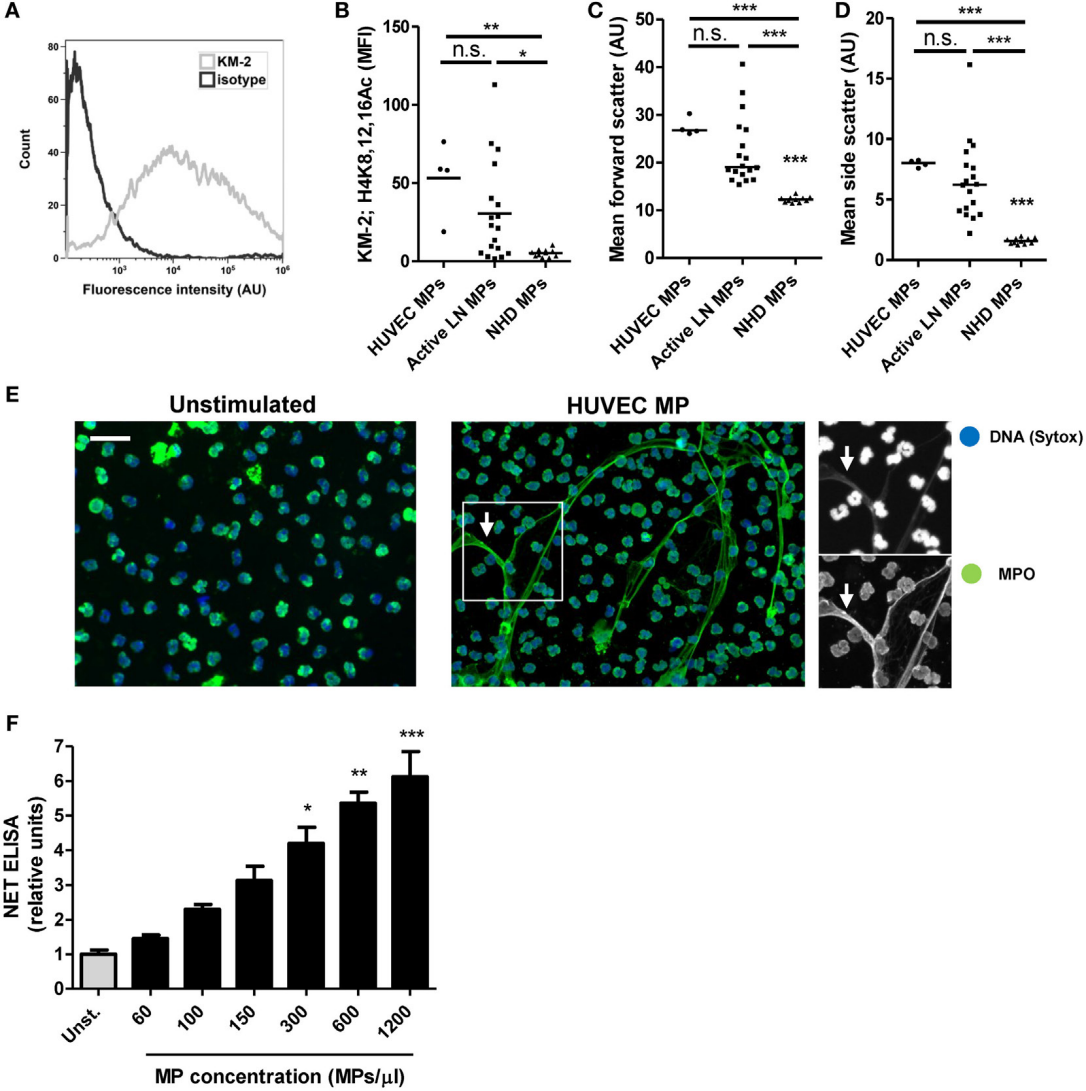
Microparticles (MPs) from apoptotic endothelial cells induce NETosis. **(A)** Human umbilical vein endothelial cell (HUVEC)-derived MPs stained for H4K8,12,16Ac (KM-2) and measured using flow cytometry corrected for isotype staining. Mean KM-2 staining **(B)**, forward scatter **(C)**, side scatter **(D)** of HUVEC-derived MPs (*n* = 4) MPs isolated from patients with active lupus nephritis (LN) (*n* = 18) and healthy donors (*n* = 11). Note that data concerning active LN patients and healthy donors are the same data as displayed in Figure 1. **(E)** Neutrophils isolated from healthy donors were either left unstimulated (left panel) or were co-incubated with HUVEC-derived MPs (right panel). Samples were fixed and stained for DNA (Sytox) and myeloperoxidase (MPO). HUVEC MPs induce the formation of neutrophil extracellular traps (NETs) identified by colocalization of MPO and DNA (right panel, arrows). **(F)** Different concentration of HUVEC-derived MPs were incubated with isolated neutrophils and NET formation was measured using a NET-specific ELISA (*n* = 4 experiments). Data are represented for each patient separately with median **(B–D)** and mean with SEM **(F)**. Scale bar: 50 µm. *p < 0.05, **p < 0.01, ***p < 0.001 compared to unstimulated control **(F)** by Student’s *t* test or compared to all other conditions **(B–D)** by one-way ANOVA with Bonferroni-corrected *post hoc* test.

We previously showed that apoptotic MPs in SLE patients predominantly originate from endothelial cells (15). Others also reported that the number of endothelial cell-derived MPs increases during active disease (32). Therefore, we used *in vitro* generated MPs derived from apoptotic HUVECs as a model to study the mechanisms involved in MP-induced NETosis. HUVEC-derived MPs contained apoptotic chromatin modification as defined by KM-2 (Figures 2A,B) and showed forward and side scatter characteristics similar to MPs isolated from patients with SLE (Figures 2C,D). HUVEC-derived MPs were internalized and induced NETosis in neutrophils isolated from healthy donors as visualized by immunofluorescence microscopy (Figure 2E; Figure S1 in Supplementary Material). Neutrophils stimulated with HUVEC-derived MPs extrude fine stretches of DNA decorated with MPO and NE (Figure 2E; Figure S2A in Supplementary Material). The induction of NETosis by HUVEC-derived MPs appeared to be concentration-dependent as quantified by NET ELISA and Sytox staining (Figure 2F; Figure S2B in Supplementary Material). Together, these data indicate that HUVEC-derived MPs can be used as a model to study MP-induced NETosis in SLE.

### HUVEC MPs Induce a Fast and ROS-Independent NETosis Mechanism

**Figure 3:**
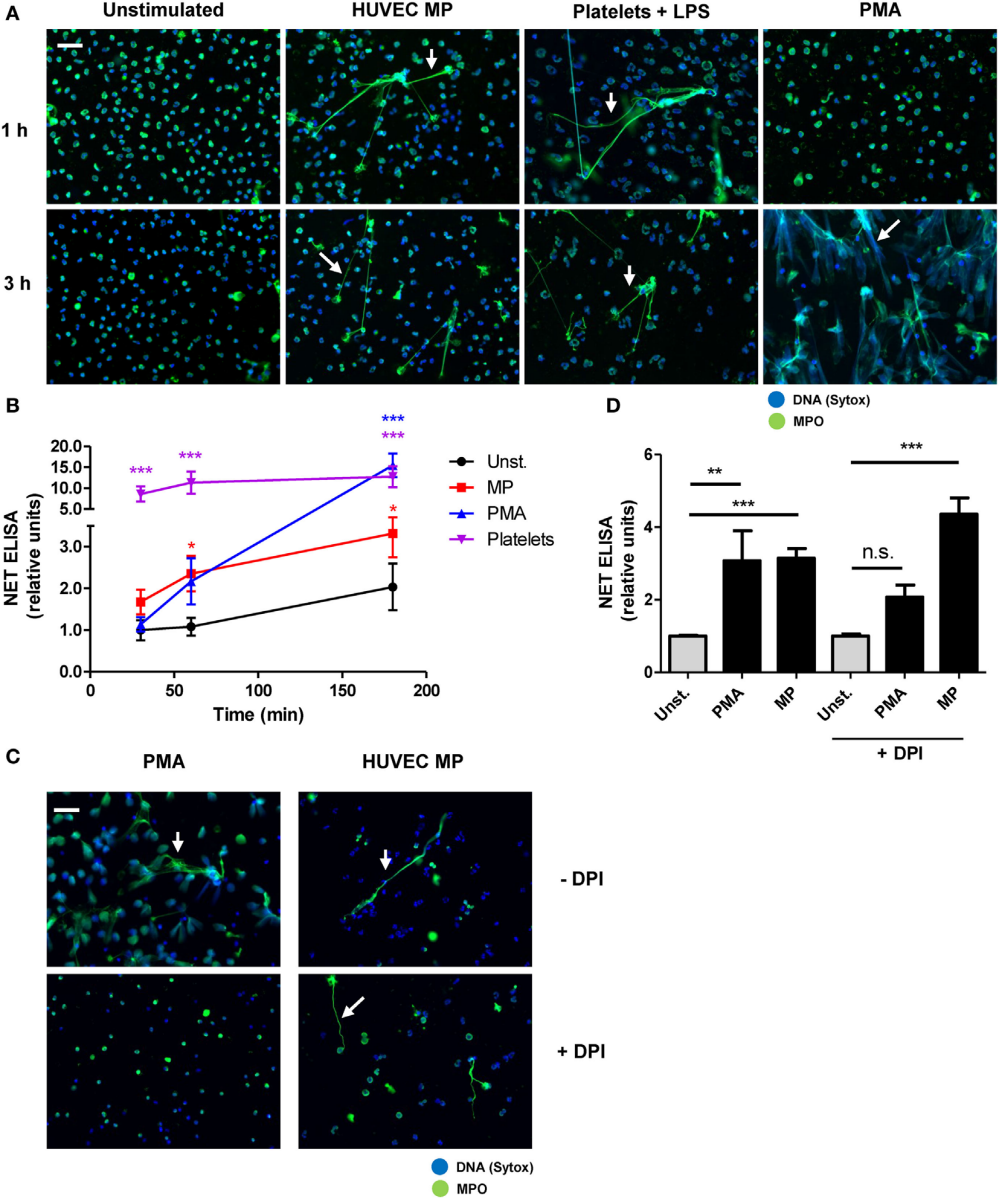
Human umbilical vein endothelial cell (HUVEC)-derived microparticles (MPs) induce a fast and ROS-independent NETosis mechanism. **(A,B)** Neutrophils were stimulated with HUVEC-derived MPs, platelets activated with lipopolysaccharide (LPS), or PMA and analyzed at different time points. **(A)** Samples were fixed and stained for myeloperoxidase (MPO) and DNA (Sytox). Arrows indicate neutrophil extracellular trap (NET) formation. **(B)** NET formation as measured using a NET-specific ELISA (*n* = 4 experiments). **(C,D)** Neutrophils were left untreated or were preincubated with diphenyleneiodonium (DPI) and subsequently stimulated with PMA or HUVEC-derived MPs for 3 h. **(C)** Immunofluorescent images stained for MPO and DNA (Sytox, arrows indicate NETs). **(D)** NETosis quantified by a NET-specific ELISA (*n* = 3 experiments). Data are presented as mean with SEM. Scale bar: 50 µm. *p < 0.05, **p < 0.01, ***p < 0.001 tested by two-way ANOVA with Bonferroni-corrected post test compared to control **(B)** one-way ANOVA followed by Bonferroni-corrected *post hoc* test **(D)**.

NETosis was initially described as a ROS-dependent cell death pathway (23), but other reports showed that NETs can also be released rapidly in a ROS-independent manner (33). For instance, activated platelets rapidly trigger ROS-independent NETosis (29, 34). We observed that HUVEC-derived MPs induced NETosis already within 30 min of incubation, with the difference compared to the control being significant from 60 min onward (Figure 3B). Over the time course of 3 h, MP-stimulated neutrophils only show a minor increase in NET production similar to the control situation. By contrast, exposure of neutrophils to PMA, a frequently used stimulus for ROS-dependent NETosis, resulted in only minor NET release after 1 h and most NETs were produced between 1 and 3 h of stimulation (Figures 3A,B; Figures S3A,B in Supplementary Material). Furthermore, exposure of neutrophils to PMA resulted in the complete rupture of nearly all neutrophils, as observed by immunofluorescence microscopy (Figure 3A), whereas HUVEC-derived MPs and LPS-activated platelets resulted in the release of NET-like structures without evidently altering neutrophil morphology (Figure 3A). Finally, inhibition of ROS by DPI could not inhibit NET release in response to HUVEC-derived MPs, whereas the release of NETs in response to PMA was reduced by DPI (Figures 3C,D; Figures S3C,D in Supplementary Material). In summary, HUVEC-derived MPs rapidly induce NETosis in a ROS-independent manner, which is fundamentally different from PMA-induced NETosis.

### Acetylated Histones Contribute to the NETosis-Inducing Capacity of MPs

**Figure 4:**
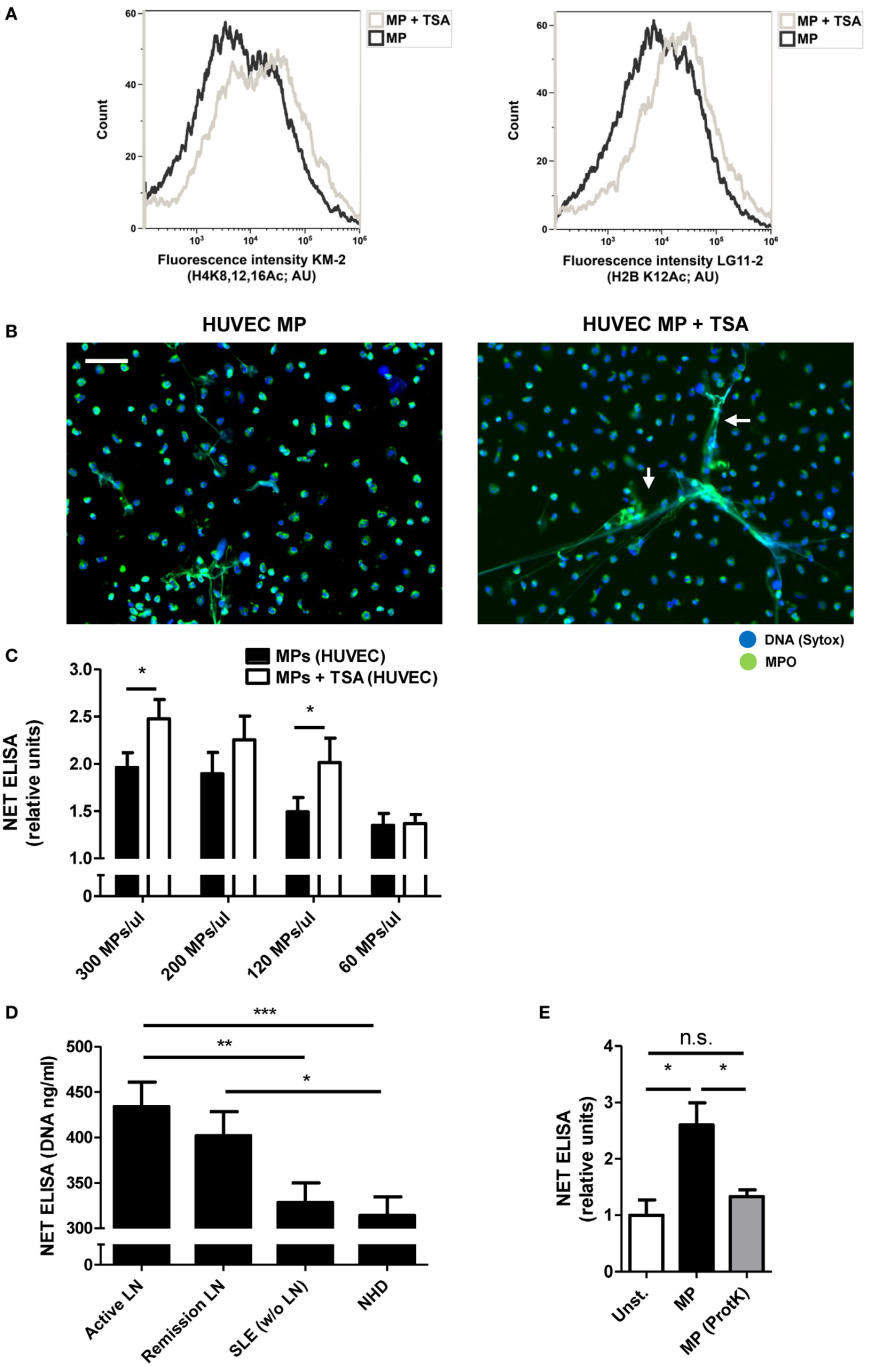
Degree of histone acetylation in microparticles (MPs) determines their NETosis-inducing capacity. **(A)** MPs isolated from human umbilical vein endothelial cell (HUVEC) treated with trichostatin A (TSA) or left untreated before induction of apoptosis stained for H4K8,12,16Ac (KM-2; left panel) and H2BK12Ac (LG11-2; right panel) and measured using flow cytometry. **(B)** MPs from TSA-treated HUVEC and normal MPs were co-incubated with isolated neutrophils and samples were stained for myeloperoxidase (MPO) and DNA [Sytox, arrows indicate neutrophil extracellular traps (NETs)]. **(C)** Different concentrations of MPs from TSA treated HUVECs and normal MPs were co-incubated with neutrophils and NETosis was measured using a NET-specific ELISA (*n* = 4). **(D)** MPs with high (*n* = 4) and low acetylation scores (KM-2, *n* = 4) in each patient group were pooled and co-incubated with neutrophils. NETs were quantified by a NET specific ELISA. **(E)** HUVEC MPs were treated with proteinase K resulting in MPs without proteins, before co-incubating with neutrophils (*n* = 3 experiments). Data are presented as mean with SEM. Scale bar: 50 µm. *p < 0.05, **p < 0.01, ***p < 0.001 determined by two-way or one-way ANOVA with Bonferroni-corrected *post hoc* test and Student’s *t* test.

Acetylated histones have immunostimulatory potential and appear to play a role in the pathogenesis of SLE (12, 28). We hypothesized that acetylated histones within apoptotic cell-derived MPs determine their potency to induce NETosis. To investigate this, we treated HUVECs with the histone deacetylase inhibitor TSA prior to the induction of apoptosis and the isolation of HUVEC-derived MPs. In this way, we generated HUVEC-derived apoptotic MPs containing increased amounts of hyperacetylated histones compared to normal apoptotic HUVEC-derived MPs (Figure 4A). Indeed, hyperacetylated MPs led to a further increase in NET production, compared to normally acetylated MPs (Figures 4B,C; Figure S4 in Supplementary Material). Plasma-derived MPs from patients with active LN displayed the highest acetylation levels (Figures 1B,C) and were more potent stimulators of NETosis compared to MPs from SLE patients without LN or MPs from NHDs (Figure 4D). In addition, ProtK-mediated degradation of all proteins, including histones, present in MPs diminished their potency to induce NETosis (Figure 4E, Figure S5 in Supplementary Material). Taken together, the presence of acetylated histones within HUVEC- or SLE-derived MPs contributes to the NETosis-inducing capacity of MPs.

### DNase Activity is Impaired in Patients with LN

**Figure 5:**
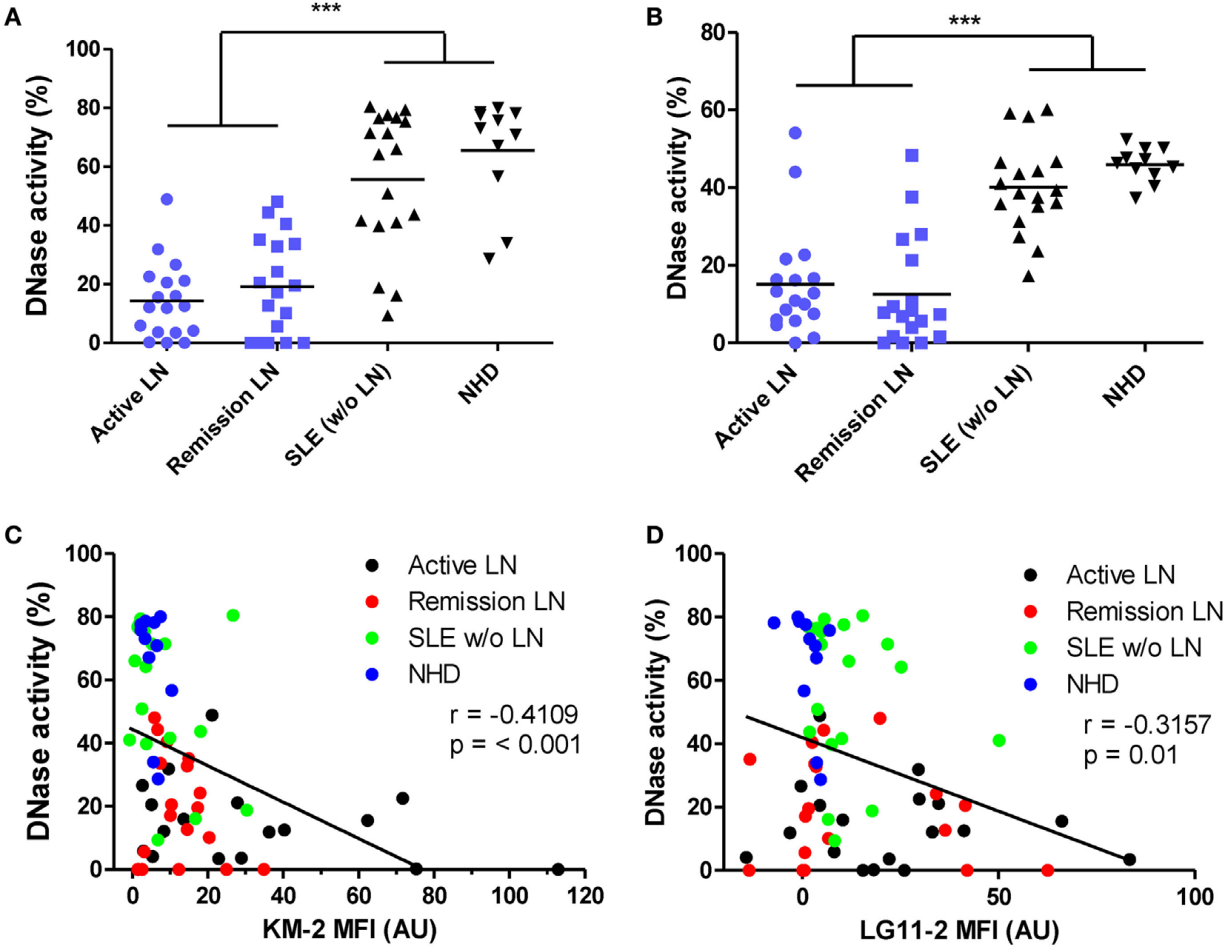
DNase activity is impaired in patients with lupus nephritis (LN). **(A,B)** Plasma of patients with active LN (*n* = 18), remissive LN (*n* = 18), systemic lupus erythematosus (SLE) patients without LN (*n* = 18), and NHD (*n* = 11) were incubated with neutrophil extracellular traps (NETs) **(A)** or DNA from calf thymus **(B)**. DNase activity is expressed as the percentage of degraded nucleic acid, measured by Sytox orange, after incubation with plasma. **(C,D)** Correlation of DNase activity on degradation of NETs with chromatin content of microparticles as measured using flow cytometry. Data are presented for each patient separately with indication of the median of the groups. ***p < 0.001 determined by one-way ANOVA followed by Bonferroni-corrected *post hoc* test. Correlations are reported as Spearman correlation coefficients.

DNase activity is important for the degradation of both NET-derived as well as MP-derived chromatin (22, 35). It appeared that patients with LN (both active and remissive) were impaired in their capacity to degrade NETs (Figure 5A) as well as nuclear DNA (Figure 5B), as has also been reported earlier (22, 36). In addition, DNase activity inversely correlated with the amount of acetylated histones in MPs (Figures 5C,D), suggesting that patients with an impaired DNase activity also fail to degrade apoptotic cell-derived chromatin *in vivo*. Altogether, these data implicate that patients with LN fail to degrade extracellular chromatin.

## Discussion

An imbalance between the formation and clearance of apoptotic cells in SLE causes the persistent presence of apoptotic debris, including cell-derived MPs, in the extracellular space. Circulating MPs in SLE patients are considered pro-inflammatory, and previously we showed that SLE-derived MPs have the capacity to enhance NETosis in blood-derived neutrophils (15). In this study, we describe that the presence of hyperacetylated chromatin in MPs is a critical determinant for the NETosis-inducing capacity of MPs in SLE patients with active LN.

A unique feature of MPs released from dying cells is that they contain nuclear components, such as histones and DNA, which carry apoptotic modifications (37). Furthermore, MPs derived from apoptotic cells tend to be bigger compared with MPs released from living cells (31). We found that MPs present in patients with active LN showed a relatively high reactivity with anti-histone antibodies recognizing apoptosis-associated histone modifications and had on average a larger forward and side scatter on flow cytometric analysis. Together, these findings suggest that apoptotic cells contribute to a considerable extent to the generation of MPs in patients with LN. The degree of apoptotic histone modifications found in MPs declined during remission of LN and was much lower in SLE patients without LN.

The presence of apoptotic cell-derived MPs in SLE patients with active LN has been linked to tissue damage induced by for example sun burns (38). In addition, apoptotic cell-derived MPs could be a reflection of an ongoing state of inflammation and tissue damage elsewhere. We propose that the presence of apoptotic cell-derived MPs in the circulation also gives rise to a pathogenic feed forward loop by interacting with circulating neutrophils and triggering NET release. Aberrant NET release and the failure of endonucleases to degrade NETs have previously been linked to SLE and LN (22, 39). We show that SLE patients with either active or remissive LN fail to degrade NET-derived chromatin, whereas SLE patients without LN are capable of degrading NETs. Thus, an impaired endonuclease activity is typical for SLE patients with LN or SLE patients with increased SLEDAI and other non-renal clinical complications (40). Undegraded NETs can ultimately deposit in the glomerular capillaries, where they may exacerbate renal inflammation by activation of complement *via* bound Ig, or by their cytotoxic histones and/or cytotoxic enzymes (e.g., MPO, NE) (41–43). This is in line with findings of circulating NET fragments and skin or renal NET depositions in lupus-prone mice and SLE patients (44), suggesting a pathogenic involvement of NETs in SLE.

A key finding of our study is that the presence of acetylated histones within the MPs is crucial for the induction of NETosis. The presence of acetylated histones within MPs inversely correlated with endonuclease activity in the plasma, suggesting that endonucleases are involved in the degradation of both NET-derived chromatin and chromatin present within apoptotic cell-derived MPs. Recent studies indicated that the failure to digest chromatin in MPs leads to autoimmunity and the induction of SLE (35). Apoptosis-associated histone modifications increase the immunostimulatory potential of histones, and anti-histone antibodies in SLE patients show an increased reactivity with modified histones compared to their unmodified counterparts (5). We previously described that hyperacetylated nucleosomes show an increased capacity to activate bone-marrow derived dendritic cells when compared to non-acetylated nucleosomes. Moreover, administration of an acetylated histone H4 peptide to lupus-prone mice enhanced proteinuria, skin lesions, and mortality, whereas its non-acetylated counterpart had not such effects (12). Acetylated histones can be sensed *via* toll-like receptors 2 and 4 (TLRs) (45), both of which are expressed by neutrophils. Therefore, it is tempting to speculate that TLR signaling is involved in MP-induced NETosis.

We found that NETosis in response to hyperacetylated histones occurred rapidly and proceeded in a ROS-independent manner. Although many NET-inducing agents typically cause ROS-dependent NETosis (46), recently a number of stimuli have been shown to promote NET formation without a requirement of ROS production (29, 34). Furthermore, lupus-prone mice deficient in ROS have markedly exacerbated lupus, including increased autoantibody profiles as well as renal disease, questioning the contribution of ROS-dependent NETosis to the pathogenesis of SLE (47). Therefore, we propose that ROS-independent NETosis driven by circulating MPs is promoting the pathogenesis of SLE.

Whether the NET release after stimulation by apoptotic cell-derived MPs has a physiological meaning is unclear. NETosis was originally described as an antimicrobial defense strategy that is triggered by molecules derived from foreign (intruding) pathogens. However, many host-derived molecules (e.g., HMGB1, uric acid) have been identified as NET-inducing stimuli (48, 49). NETs can also function as a transient barrier during tissue injury, a process in which debris released from dying cells triggers NETosis and the aggregation of NETs, thereby isolating injured tissue and limiting the spread of cell death-associated pro-inflammatory mediators (50, 51). It remains speculative that the MP-induced NETosis finds it origin in aforementioned mechanism.

**Figure 6:**
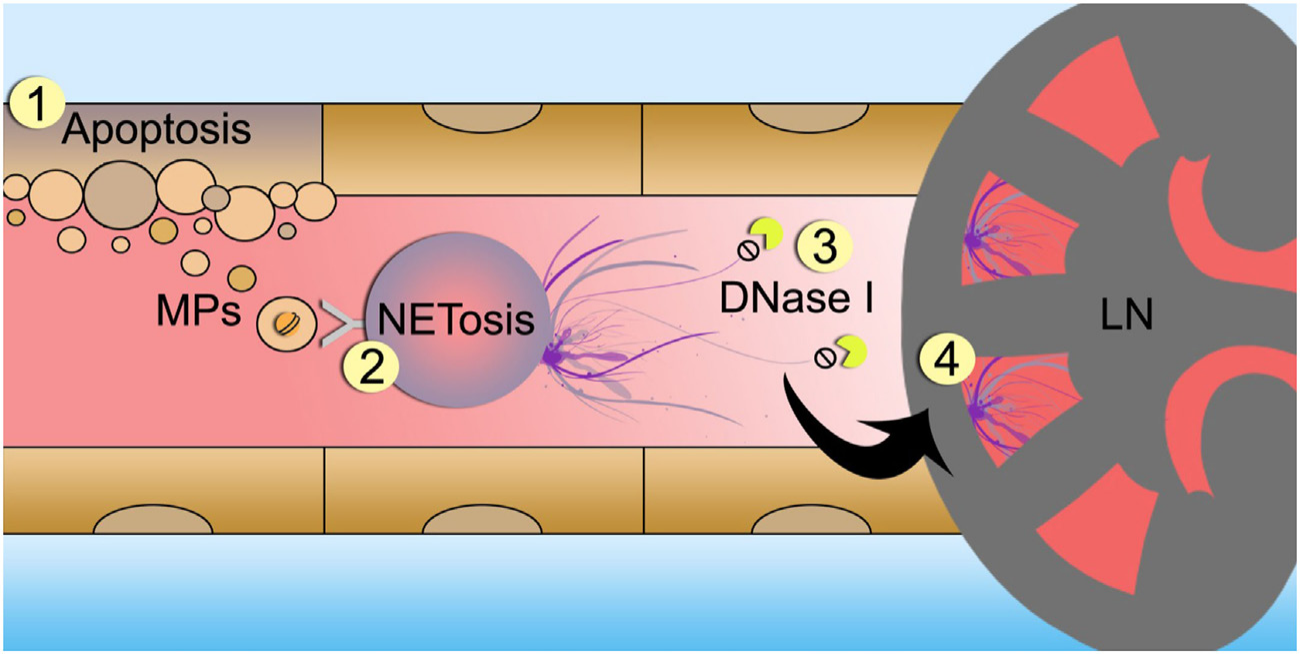
Schematic overview of proposed mechanism of microparticle (MP)-induced NETosis in patients with lupus nephritis (LN). Cells undergoing apoptosis release MPs containing hyperactylated histones (1). In patients with LN MPs accumulate in the circulation and trigger neutrophils to undergo NETosis (2). Since DNase activity is impaired in LN patients, neutrophil extracellular traps (NETs) are not properly degraded (3). NETs deposit in the glomerular capillaries and induce tissue injury and inflammations leading to nephritis and further cell death (4).

In conclusion, we show that MPs induce the formation of NETs in SLE patients with LN. The degree of acetylated chromatin present in the MPs determines their potency to stimulate neutrophils to go into NETosis. We propose that during active LN, apoptotic cell-derived MPs are formed. Without the proper functioning of endonucleases, MPs enriched in apoptosis-associated histone modifications persist within the circulation, thereby triggering NETosis and further kidney damage when the formed NETs deposit in the renal capillaries. This causes further release of apoptotic cell-derived MPs, thereby establishing a vicious pathogenic circle (Figure 6).

## Ethics Statement

This study was carried out in accordance with the recommendations of the Radboudumc ethics committee with written informed consent from all subjects. All subjects gave written informed consent in accordance with the Declaration of Helsinki. The protocol was approved by the Radboudumc ethics committee.

## Author Contributions

NR designed and performed research, analyzed and interpreted data, and wrote the manuscript; EP interpreted data and wrote the manuscript; JL performed research and analyzed data; LH interpreted data and wrote the manuscript; JV designed and supervised research, interpreted data, and wrote the manuscript.

## Conflict of Interest Statement

The authors declare that the research was conducted in the absence of any commercial or financial relationships that could be construed as a potential conflict of interest.
