# Supplementary material for: Acetylated Histones in Apoptotic Microparticles Drive the Formation of Neutrophil Extracellular Traps in Active Lupus Nephritis
Source: Front Immunol. 2017 Sep 14;8:1136. doi: 10.3389/fimmu.2017.01136 (PMC5604071; doi:10.3389/fimmu.2017.01136)
Supplement: Supplementary file 1 [file Data_Sheet_1.DOCX]

Supplementary Material

Acetylated Histones in Apoptotic Microparticles drive the Formation of Neutrophil Extracellular Traps in active Lupus Nephritis

Nils Rother^1^, Elmar Pieterse^1^, Jelle Lubbers^1^, Luuk Hilbrands^1^, Johan van der Vlag^1*^

^1^ Department of Nephrology, Radboud Institute for Molecular Life Sciences, Radboud University Medical Center, Nijmegen, The Netherlands.

*** Correspondence:**Dr. J. van der Vlag, Nephrology Research Laboratory (480), Department of Nephrology, Radboud University Medical Center, Geert Grooteplein 10, 6525 GA Nijmegen, The Netherlands, Tel: +31-24-3616539, E-mail: [johan.vandervlag@radboudumc.nl](mailto:johan.vandervlag@radboudumc.nl)

# Supplementary Data

## Supplementary Material and Methods

**SDS-PAGE gel, Silver staining and Western blot analysis**

SDS-PAGE and Silver staining was performed as described previously (Blum et al., 1987). For Western blot analysis, proteins were transferred onto nitrocellulose blotting membranes (GE Healthcare Life Science). Blots were blocked with blocking reagent (Roche) and incubated with KM-2(Dieker et al., 2007), followed by a goat-anti-mouse peroxidase labeled secondary antibody (Jackson Immuno Research). Blots were developed using WesternBright^TM^ Quantum detection kit (Advansta) according to manufacturer’s instructions. Images were obtained using ChemiDoc^TM^ XRS+ imaging system (Bio-rad).

**Confocal microscopy**

HUVEC were stained with 10µM PKH26 (Thermo Scientific) according to manufacturer’s instructions. Apoptotic microparticles were induced and collected as described above. Neutrophils were co-incubated with PKH-labelled MPs for 3h before cells were fixed and stained with phalloidin-FITC as a background staining for the neutrophils and DAPI. Slides were embedded in Vectashield medium (Brunschwig Chemie) and imaged with an Olympus FV1000 Confcocal Laser Scanning microscope.

## Supplementary References

Blum, H., Beier, H., Gross, H.J. (1987). Improved silver staining of plant proteins, RNA and DNA in polyacrylamide gels. *Electrophoresis* 8, doi:

Dieker, J.W., Fransen, J.H., van Bavel, C.C., Briand, J.P., Jacobs, C.W., Muller, S., et al. (2007). Apoptosis-induced acetylation of histones is pathogenic in systemic lupus erythematosus. *Arthritis Rheum* 56,1921-1933, doi: 10.1002/art.22646.

# Supplementary Figures and Tables

## Supplementary Figures

**Supplementary Figure 1: Microparticles are internalized by neutrophils.** MP from PKH stained HUVEC cells (red) were co-incubated with CFSE-labelled neutrophils (green). Cells were fixed and stained with DAPI (blue). Cells were imaged with confocal microscope at different focal planes creating a z-stack. White lines indicate corresponding points in the orthogonal planes (right and lower panel). Scale bar: 5µm.

**Supplementary Figure 2: Microparticles from apoptotic endothelial cells induce NETosis.** (**A**) Neutrophils isolated from healthy donors were either left unstimulated (left panel) or were co-incubated with HUVEC-derived MPs (right panel). Samples were fixed and stained for DNA (Sytox) and neutrophil elastase (NE). HUVEC MPs induce the formation of NETs identified by colocalization of MPO and DNA (single staining, small insert) (**B**) Different concentration of HUVEC-derived MPs were incubated with isolated neutrophils and extracellular DNA measured using Sytox orange (n=4 experiments). Scale bar: 50µm. * p<0.05, ** p<0.01, compared to unstimulated control by Students t test.

**Supplementary Figure 3: HUVEC-derived MPs induce a fast and ROS independent NETosis mechanism.** (**A, B**) Neutrophils were stimulated with HUVEC-derived MPs, platelets activated with LPS or PMA and analyzed at different time points. (**A**) Samples were fixed and stained for NE and DNA (Sytox). Arrows indicate NET formation. (**B**) Extracellular DNA was measured using Sytox orange (n=4 experiments). (**C, D**) Neutrophils were left untreated or were preincubated with DPI and subsequently stimulated with PMA or HUVEC-derived MPs. (**C**) Immunofluorescent images stained for NE and DNA (Sytox). (**D**) Extracellular DNA quantified using Sytox orange (n=4 experiments). * p<0.05, ** p<0.01, *** p<0.001 tested by two-way ANOVA followed by Bonferroni corrected post test (in **B**) and tested by one-way ANOVA followed by Bonferroni corrected post-hoc test (in **D**).

**Supplementary Figure 4: Hyperactylated MPs induce higher levels of NETosis.** MPs from TSA treated HUVEC and normal MPs were co-incubated with isolated neutrophils and samples were fixed and stained for NE and DNA (Sytox).

**Supplementary Figure 5: Proteinase K treatment degrades histones present in MPs.** HUVEC-derived MPs were treated with proteinase K and loaded on a SDS-PAGE gel together with untreated MPs. Proteins were analyzed by Silver staining (**A**) or blotted on a nitrocellulose membrane and stained with KM-2 (**B**).

## Supplementary Tables

**Supplementary Table 1: Patient characteristics**

|  | **Active** | **Remission** | **SLE w/o LN** | **p-value** |
| --- | --- | --- | --- | --- |
| **Number** | 18 | | 18 | - |
| **Age (mean ± SD)** | 32.3 (10.6) | | 57.1 (15.6) | - |
| **Gender female (n,%)** | 15 (83%) | | 15 (83%) | - |
| **Time to remission (weeks)** | 43 [18;90] | | - | - |
| **C3 (g/l)** | 0.525 [0.35;0.66] | 0.755 [0.68;0.84] | - | <0.001 |
| **Anti-dsDNA (IU/ml)** | 85.5 [20.5;637.5] | 17.5 [7;60.75] | - | <0.05 |
| **Creatinin (µmol/l)** | 108.5 [84.7;157.2] | 79.5 [71;92.25] | - | <0.05 |
| **Proteinuria (g/24h)** | 3.28 [1.85;4.79] | 0.4 [0.28;0.5] | - | <0.0001 |
| **SLEDAI** | 15 [12;21] | 4 [2.25;6] | 0.66 [0;1] | <0.0001 |

C3, complement C3; Anti-dsDNA, anti double stranded DNA antibodies; SLEDAI, SLE disease activity index. Data are given as median and interquartile ranges or as mean ± SD were indicated. P-value for paired t-test between Active and Remission group.
