# Supplementary figures and images for: Acetylated Histones in Apoptotic Microparticles Drive the Formation of Neutrophil Extracellular Traps in Active Lupus Nephritis
Source: Front Immunol. 2017 Sep 14;8:1136. doi: 10.3389/fimmu.2017.01136 (PMC5604071; doi:10.3389/fimmu.2017.01136)

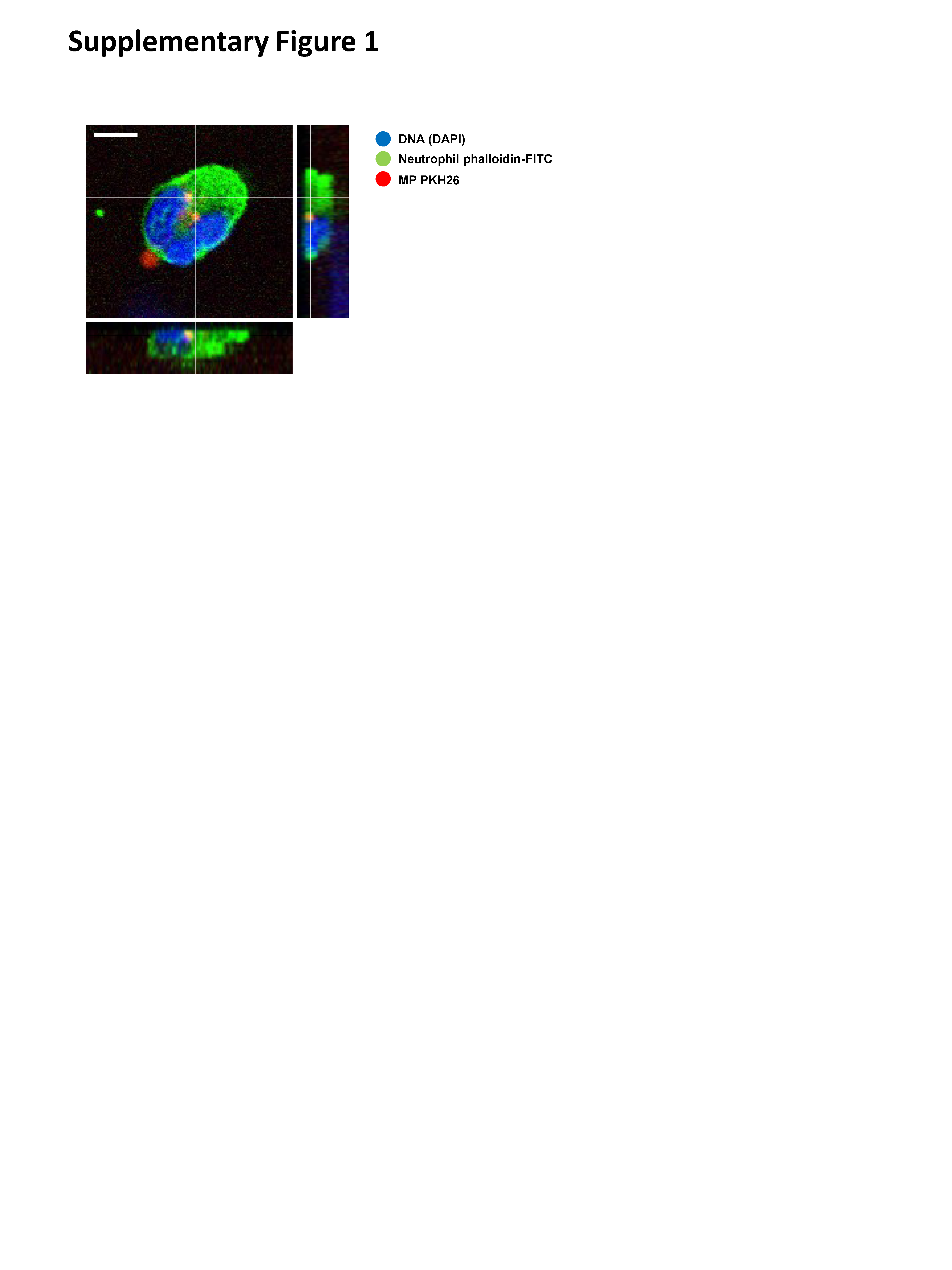

Supplement: Supplementary file 2 [file Image_1.TIFF]

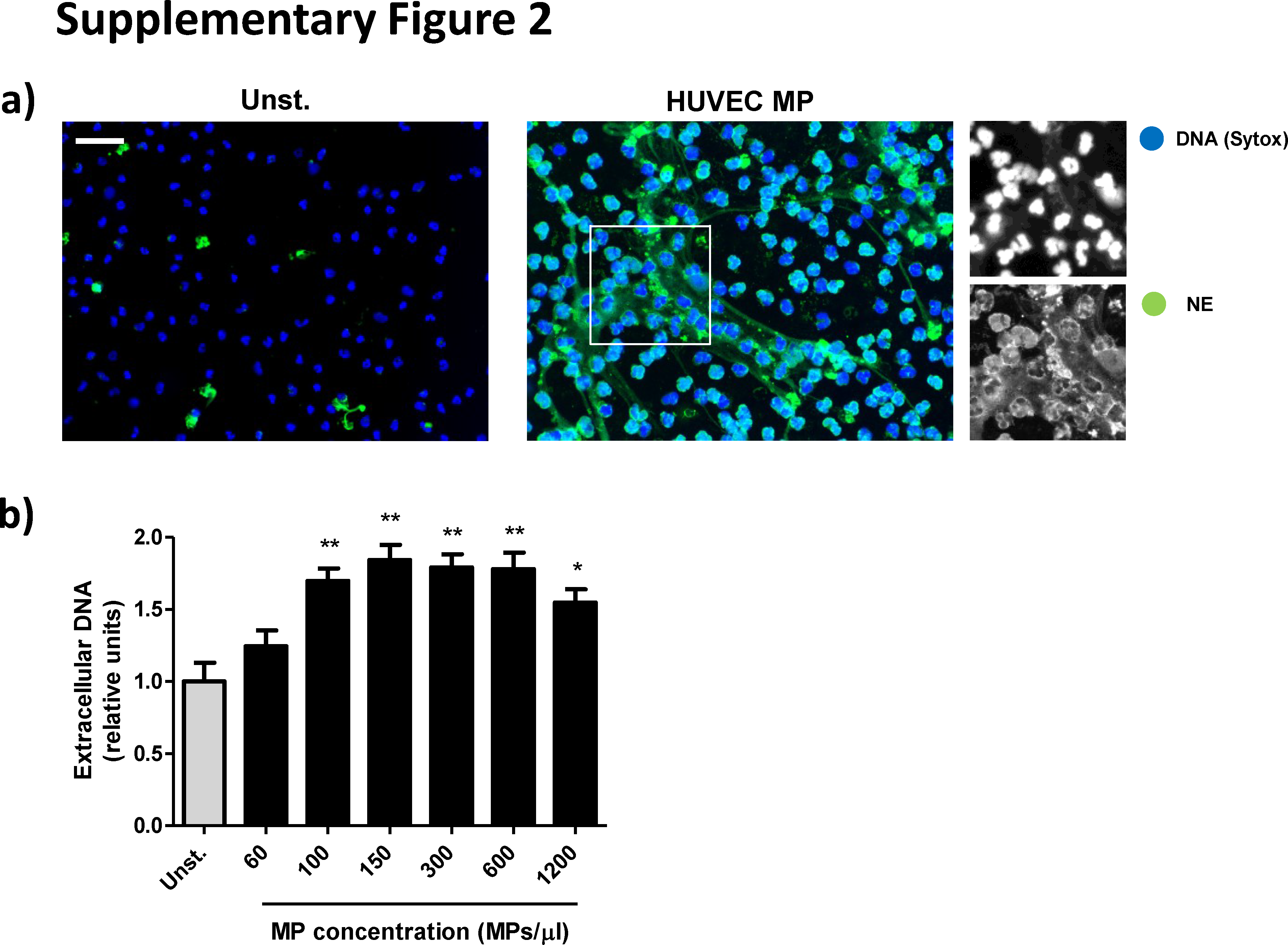

Supplement: Supplementary file 3 [file Image_2.TIFF]

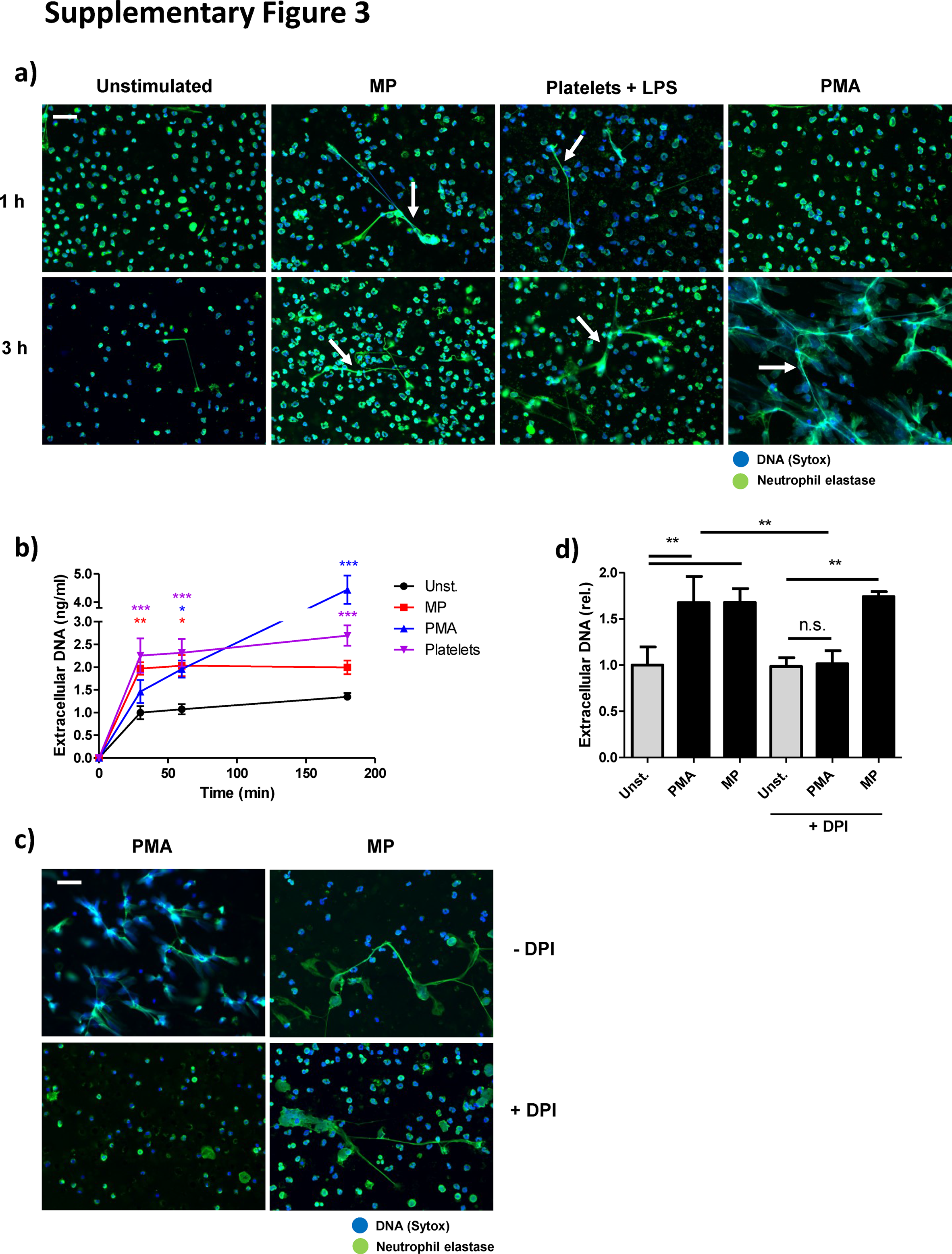

Supplement: Supplementary file 4 [file Image_3.TIF]

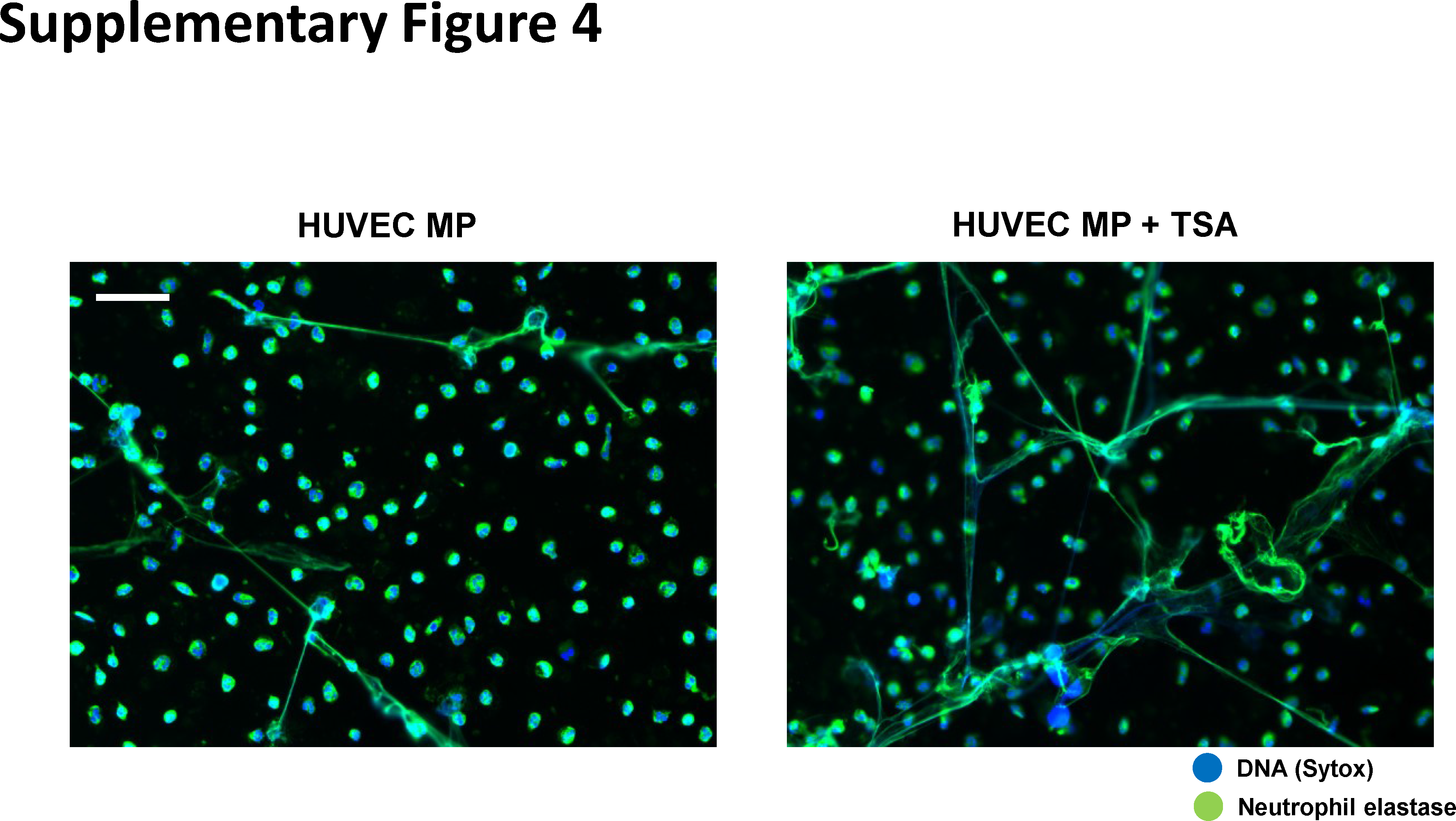

Supplement: Supplementary file 5 [file Image_4.TIFF]

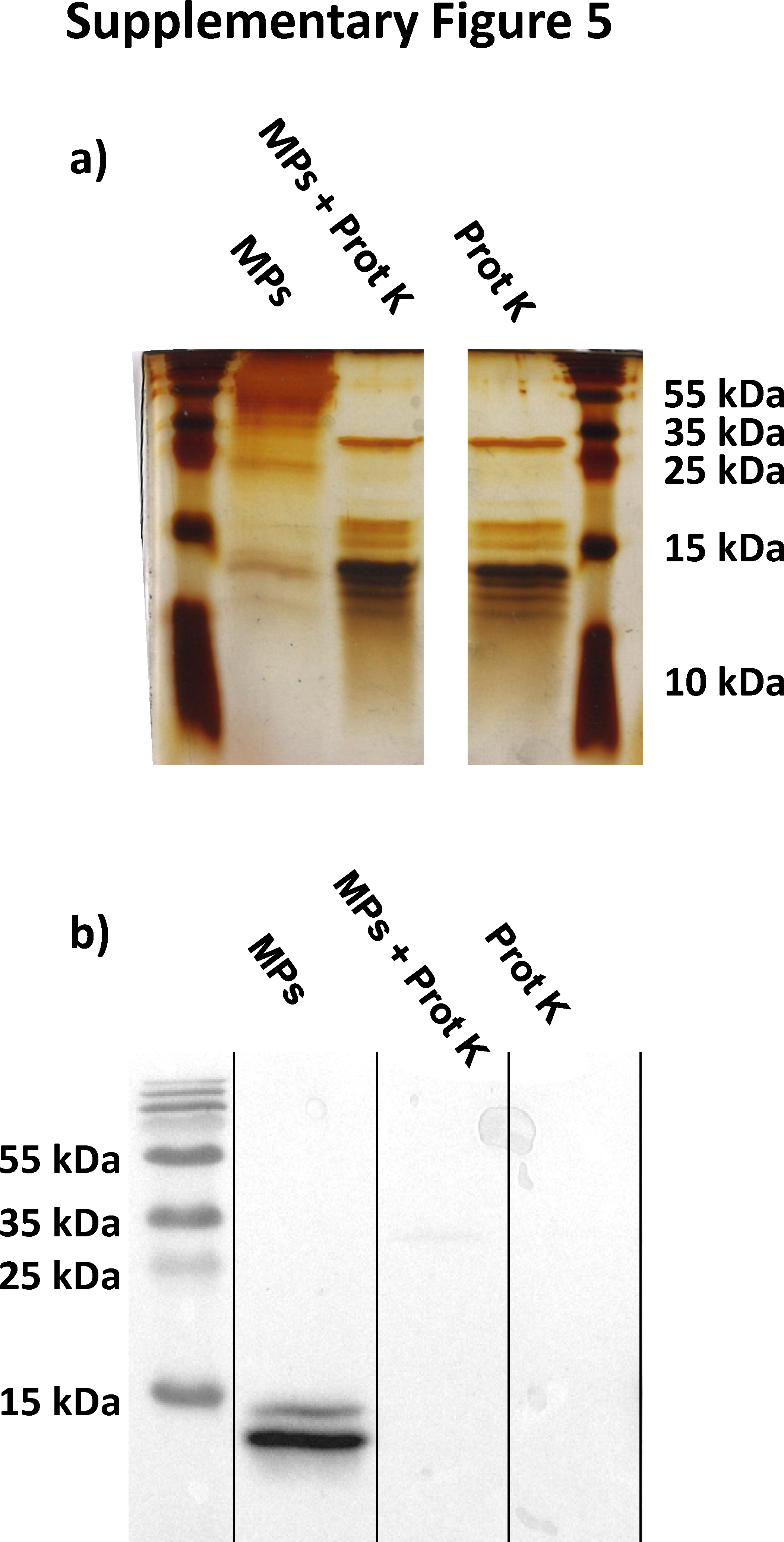

Supplement: Supplementary file 6 [file Image_5.TIF]
